# Exploration of key ferroptosis-related genes and immune infiltration in Crohn’s disease using bioinformatics

**DOI:** 10.1038/s41598-023-40093-w

**Published:** 2023-08-07

**Authors:** Xiaoting Tang, Weitao Hu, Wei You, Taiyong Fang

**Affiliations:** 1 Department of Gastroenterology, The Second Affiliated Hospital of Fujian Medical University, 34 North Zhongshan Road, Licheng District, Quanzhou, 362000 Fujian People’s Republic of China; 2 Department of Rheumatology, The Second Affiliated Hospital of Fujian Medical University, Quanzhou, 362000 Fujian People’s Republic of China; 3 Department of Neurosurgery, Zhangzhou Municipal Hospital of Fujian Province and Zhangzhou Affiliated Hospital of Fujian Medical University, Zhangzhou, 363000 Fujian Province People’s Republic of China

**Keywords:** Immunology, Gastroenterology

## Abstract

Crohn's disease (CD) is a type of inflammatory bowel disease (IBD) that manifests mainly as chronic inflammation in different parts of the gastrointestinal tract, and its incidence has come to be increasing in recent years. Ferroptosis, a novel type of programmed cell death, it seems the role of ferroptosis-related biomarkers in CD has not been mentioned. Thus, the role of ferroptosis in CD and its relationship with immune infiltration were explored in this study. The CD dataset was downloaded from the Gene Expression Omnibus database. The validated ferroptosis genes (FRGs) were retrieved from the public FerrDb database. The gene expression matrix of the CD dataset was analyzed with the “limma” package in R language to obtain differentially expressed genes (DEGs) between diseased and healthy samples. Then, intersecting genes between DEGs and FRGs were identified as differentially expressed ferroptosis-associated genes (DE-FRGs). Protein–protein interaction (PPI) network analysis and visualization were carried out with STRING and Cytoscape, and key CD ferroptosis-related genes (CD-FRGs) were identified along with their Gene Ontology (GO) and Kyoto Encyclopedia of Genes and Genomes (KEGG) pathways using the clusterProfiler package. Immune cell infiltration was analyzed with CIBERSORT. The correlation between key CD-FRGs and immune-infiltrated cells in CD was studied by Spearman's correlation method. A total of 37 DE-FRGs and 6 key CD-FRGs (*CAV1, CD44, HIF1A, IFNG, TIMP1* and *TLR4*) were identified. GO and KEGG functional analysis indicated these genes enrichment in programmed cell death and apoptotic process, HIF-1 signaling pathway and IBD. Infiltration matrix analysis of immune cells showed abundant T cells CD4 memory activated, M1 macrophages, M2 macrophages, Mast cells activated and Neutrophils in CD intestinal tissues. The 6 key CD-FRGs were correlated with immune-infiltrated cells in CD based on correlation analysis. Taken together, immune cells with abnormal infiltration can be implicated in CD due to ferroptosis. This study identified 6 key CD-FRGs that may be key biomarkers of ferroptosis in CD; they include *CAV1, CD44, HIF1A, IFNG, TIMP1* and *TLR4*. These findings suggest that the immune response is critical in CD caused by ferroptosis through the interaction between key CD-FRGs and immune infiltrating cells.

## Introduction

Crohn's disease (CD) is a type of inflammatory bowel disease (IBD) that manifests mainly as chronic inflammation in different parts of the gastrointestinal tract, and its incidence has come to be increasing in recent years^1^. However, the etiology of CD remains unclear, and it has been shown that genetic, immunological and environmental factors increase the risk of its development and progression^2^. The disease course of CD is progressive and devastating, with not only intestinal destruction but also extraintestinal systemic manifestations, which will seriously affect the quality of life and prognosis of patients^3^. In the last two decades, the use of biologics such as infliximab, adalimumab and vedolizumab has greatly expanded the therapeutic options for CD^4,5^. Even though these therapies account for significant advances in the treatment of CD, there are still several patients that are not sensitive to these agents (e.g., anti-TNF antibodies)^6^. Notably, specific biomarkers can help clinicians benefit in the diagnosis and intervention of CD, as well as play a role in clarifying treatment response and predicting CD relapse. Therefore, it is important to study and explore the precise molecular mechanisms and markers of CD for the development of its therapeutic strategies.

It has been demonstrated in many studies that the progression of IBD is associated with cell death. A growing body of evidence suggests that programmed cell death, including apoptosis, necrosis, and autophagy, is involved in the progression of CD in various ways^7–10^. Ferroptosis is a newly discovered regulated form of cell death in recent years, which is driven by the lethal build-up of lipid peroxides catalyzed with cellular free iron^11^. The essential features of Ferroptosis, including iron deposition, glutathione (GSH) depletion, glutathione peroxidase 4 (GPX4) deactivation, and lipid peroxidation, have been found in abundance in the damaged gastrointestinal tract of patients with IBD^10^. Xu et al. found that Ferrostatin-1, a ferroptosis inhibitor, attenuated the pathological changes of CD-like colitis induced by TNBS in mice^12^. It has been shown that abnormal ferroptosis has been detected in CD^13^. Of interest, in terms of dietary habits, it has been shown that dietary polyunsaturated fatty acid (PUFA) is a trigger for GPX4 (which prevents ferroptosis) to limit the inflammatory phenotype of human CD mucosa^14^. However, studies on ferroptosis in CD are still relatively few and, furthermore, it seems that the role of ferroptosis-related biomarkers in CD has not been mentioned so far.

To investigate whether or how ferroptosis is involved in the progression of CD, microarray data were collected from the GEO database with intestinal tissues of CD patients and healthy individuals. Subsequently, CD-related ferroptosis genes were screened by bioinformatics methods and immuno-infiltration analysis was performed to analyze their correlation. Finally, 37 differentially expressed ferroptosis-related genes (DE-FRGs), and 6 key CD ferroptosis-related genes (CD-FRGs) were identified as potential ferroptosis-related biomarkers of CD.

## Materials and methods

### Data acquisition

The Gene Expression Omnibus (GEO; www.ncbi.nlm.nih.gov/geo/)^15^ database was used to search gene microarray data of CD intestinal tissue samples. The GSE16879^16^ dataset, containing 12 healthy controls and 37 CD intestinal tissue samples was selected. Probes were converted to gene symbols according to the GPL570 platform ([HG-U133_Plus_2] Affymetrix Human Genome U133 Plus 2.0 Array). Ferroptosis-Related Genes (FRGs) that drive, suppress, or mark ferroptosis were retrieved from the public FerrDb database (http://www.zhounan.org/ferrdb)^17^. The final 365 FRGs obtained after removing duplicate genes were used for subsequent analysis. Two microarray datasets of CD (GSE59071^18^: 8 active CD vs. 11 healthy controls; GSE95095: 24 active CDs vs. 12 healthy controls) were used to validate the expression of key CD-FRGs. Fold changes greater than 2 or less than 0.5 were considered to be significantly differentially expressed. The *p*-value was corrected by controlling the false discovery rate (FDR), and the adjusted *p*-value (Q-value = FDR) was taken to be < 0.05. The "limma" package (http://www.bioconductor.org/packages/release/bioc/html/limma.html) was also applied to identify DEGs with a threshold of |log2 fold change (FC)|≥ 1, adj. *P-value (FDR)* < 0.05. Results are shown as volcano plots with the key gene labeled.

### Identification of differentially expressed ferroptosis-related genes (DE-FRGs)

Analysis of the gene expression matrix from the GSE16879 dataset was performed using the "limma" package (http://www.bioconductor.org/packages/release/bioc/html/limma.html) in R language to obtain DEGs between CD and healthy samples. In addition, |log2 FC|> 1 and adj. *P-value (FDR)* < 0.05 were set as the selection criteria for DEGs. Subsequently, the overlapping genes between DEGs and FRGs were identified as differentially expressed ferroptosis-related genes (DE-FRGs).

### Protein–protein interaction (PPI) network construction and module analysis

Analyses of the interactions between different DE-FRGs were performed using the STRING database (http://string-db.org/)^19^. The PPI network was built and visualized with Cytoscape software 3.9.1. (http://cytoscape.org/)^20^. The key module in PPI network was identified using the Cytoscape plugin Molecular Complex Detection (MCODE) (version 2.0), a plug-in for clustering based on the topology of a given network to identify densely connected regions^21^. The overlap of the top 10 genes of the MCC, Maximum Neighborhood Component (MNC), DNMC, Closeness, Degree and Edge Infiltration Component (EPC) algorithms were identified as key Crohn's disease ferroptosis-related genes (CD-FRG) using Cytoscape's plug-in cytoHubba.

### Functional enrichment of DE-FRGs and hub CD-FRGs

Using the clusterProfiler package(https://www.bioconductor.org/packages/release/bioc/html/clusterProfiler.html) in R language to identify gene ontology (GO) and Kyoto Encyclopedia of Genes and Genomes (KEGG)^22–24^ pathways to describe the characteristics of DE-FRG and key CD-FRG and to explore their underlying biological processes (BP), cellular components (CC), molecular functions (MF) and important signaling pathways. The smallest gene set was chosen as 5 and the largest as 5000. *P* < 0.05 and false detection rate < 0.1 were considered statistically significant.

### Evaluation of subtype distribution among immune-infiltrated cells

A normalized gene expression matrix was shown to be transformed by CIBERSORT into a composition of 22 immune cell types based on a deconvolution algorithm^25^. In this study, CIBERSORT was used to calculate the composition of immune cells in CD and healthy samples. The algorithm used the LM22 signature and performed 1000 permutations.

### Analysis of correlations and differences between immune-infiltrated cells

For the assessment of the correlation between different immune cells, Pearson correlation coefficient was obtained from the sample data screened by CIBERSORT, *p* < 0.05. A rank sum test was used to compare the CD group with the control group.

### Correlation between key CD-FRGs and immune-infiltrated cells in CD

Spearman correlation analysis was performed between the component profiles of GSE16879 immune-infiltrated cells analyzed by CIBERSORT and the gene expression profiles of this dataset. Screening and determination of correlation between key CD-FRG and immune-infiltrated cells using Spearman’s correlation coefficient (r) > 0.6 and *p* < 0.05.

### Statistical analysis

Statistical analysis and graphs were performed using Sangerbox online software (http://sangerbox.com/) and GraphPad Prism 6.0 software. Fold changes greater than 2 or less than 0.5 were considered to be significantly differentially expressed. The *p*-value was corrected by controlling the false discovery rate (FDR), and the adjusted *p*-value (Q-value = FDR) was taken to be < 0.05. Correlation coefficient r-values > 0.5 were considered to have moderate and above correlation and *p* < 0.05.

## Results

### Identification of DE-FRGs in CD

**Figure 1:**
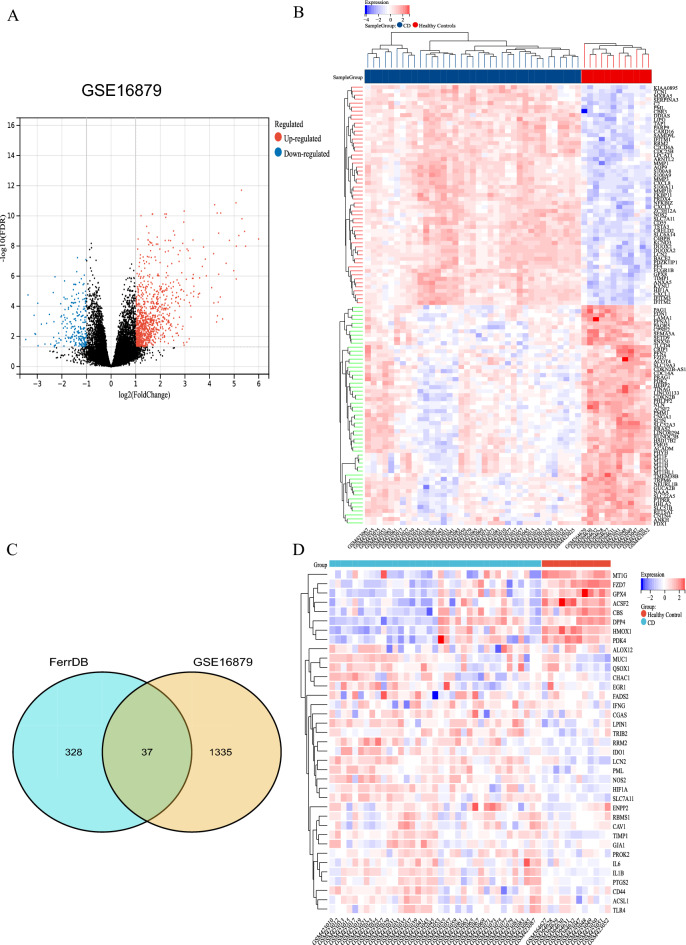
Screening of Differentially Expressed Ferroptosis-Related Genes (DE-FRGs). (**A**) Volcano plots showing significantly differentially expressed genes. |Log2FC|≥ l and *P* < 0.05 were taken for filtering. Blue dots indicate genes significantly down-regulated, red dots indicate genes significantly up-regulated, and black dots indicate genes without any significant differences shown. (**B**) Heatmap showing the expression of DEGs in GSE16879. Blue indicates lower gene expression and red indicates higher gene expression. (**C**) Display of DE-FRG by Venn diagram. (**D**) Heatmap showing the expressions of the 37 DE-FRGs in CD dataset GSE16879.

The GSE16879 dataset was retrieved from the GEO database, which contained gene expression profiles from 12 healthy intestinal and 37 CD intestinal biopsy tissue samples. After normalization of microarray results, 1372 DEGs were identified, including 1082 up-regulated genes and 290 down-regulated genes (Fig. 1A,B). A total of 365 FRGs with experimentally validated FRGs were retrieved from FerrDb, a database of regulators, markers and diseases implicated in ferroptosis. After overlapping DEGs and FRGs to take the intersection, a total of 37 FRGs were identified as DE-FRGs (Fig. 1C). Figure 1D showed the expression of 37 DE-FRGs in the CD dataset GSE16879.

### Construction of PPI network, analysis of modules and identification of key CD-FRGs

**Figure 2:**
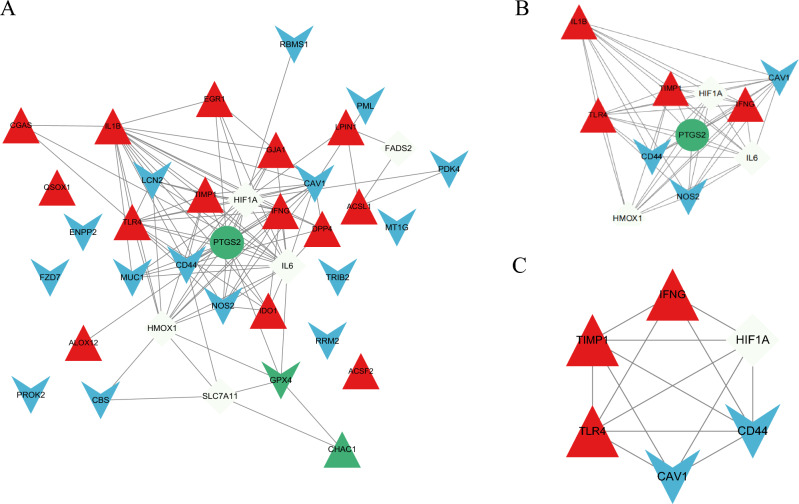
Construction and module analysis of PPI network. (**A**) PPI network of DE-FRGs (37 nodes and 106 edges). (**B**) Identification of the most densely connected regions (11 nodes and 51 edges) in PPI network through MCODE. (**C**) Identification of key CD-FRGs by 6 algorithms of cytoscape plugin cytoHubba. Red triangle indicates the driver of ferroptosis, blue V indicates the suppressor of ferroptosis, green circle indicates the marker of ferroptosis, green triangle both driver and marker of ferroptosis, green V indicates both suppressor and marker of ferroptosis, white diamond indicates the both driver and suppressor of ferroptosis.

The PPI construction for DE-FRGs was performed with the STRING database and the results were subsequently visualized with Cytoscape (Fig. 2A). The MCODE plug-in recognized the most densely connected regions (11 nodes and 51 edges) of the PPI network (Fig. 2B). The top 10 genes were obtained by 6 algorithms of Cytoscape plug-in cytoHubba (Table 1). Subsequently, the final six crossover genes obtained by overlapping the results derived from the MCC, DNMC, MNC, Degree, Closeness and EPC algorithms were chosen as the key CD-FRGs, which included *CAV1, CD44, HIF1A, IFNG, TIMP1* and *TLR4* (Fig. 2C). Of note, all these key CD-FRGs were upregulated in patients with CD. Table 2 indicated the details of these 6 key CD-FRGs.

**Table 1:** The top 10 hub genes rank in cytoHubba.

MCC	DNMC	MNC	Degree	EPC	Closeness	Overlap
CAV1	CAV1	CAV1	CAV1	CAV1	CAV1	CAV1
CD44	CD44	CD44	CD44	CD44	CD44	CD44
HIF1A	GJA1	HIF1A	HIF1A	HIF1A	HIF1A	HIF1A
HMOX1	HIF1A	HMOX1	HMOX1	HMOX1	HMOX1	IFNG
IFNG	IDO1	IFNG	IFNG	IFNG	IFNG	TIMP1
IL1B	IFNG	IL1B	IL1B	IL1B	IL1B	TLR4
IL6	LCN2	IL6	IL6	IL6	IL6	
PTGS2	NOS2	PTGS2	PTGS2	PTGS2	PTGS2	
TIMP1	TIMP1	TIMP1	TIMP1	TIMP1	TIMP1	
TLR4	TLR4	TLR4	TLR4	TLR4	TLR4

**Table 2:** The top 6 hub genes and their functions.

Gene symbol	Description	Function
CAV1	Caveolin-1	May act as a scaffolding protein within caveolar membranes. Interacts directly with G-protein alpha subunits and can functionally regulate their activity
CD44	CD44 antigen	Receptor for hyaluronic acid (HA). Adhesion with HA plays an important role in cell migration, tumor growth and progression
HIF1A	Hypoxia-inducible factor 1-alpha	Functions as a master transcriptional regulator of the adaptive response to hypoxia
IFNG	Interferon gamma	Produced by lymphocytes activated by specific antigens or mitogens. IFN-gamma, in addition to having antiviral activity, has important immunoregulatory functions
TIMP1	Metalloproteinase inhibitor 1	Metalloproteinase inhibitor that functions by forming one to one complexes with target metalloproteinases, such as collagenases, and irreversibly inactivates them by binding to their catalytic zinc cofactor
TLR4	Toll-like receptor 4	Cooperates with LY96 and CD14 to mediate the innate immune response to bacterial lipopolysaccharide (LPS). Acts via MYD88, TIRAP and TRAF6, leading to NF-kappa-B activation, cytokine secretion and the inflammatory response

### Validation of the key CD-FRGs expression in CD

**Figure 3:**
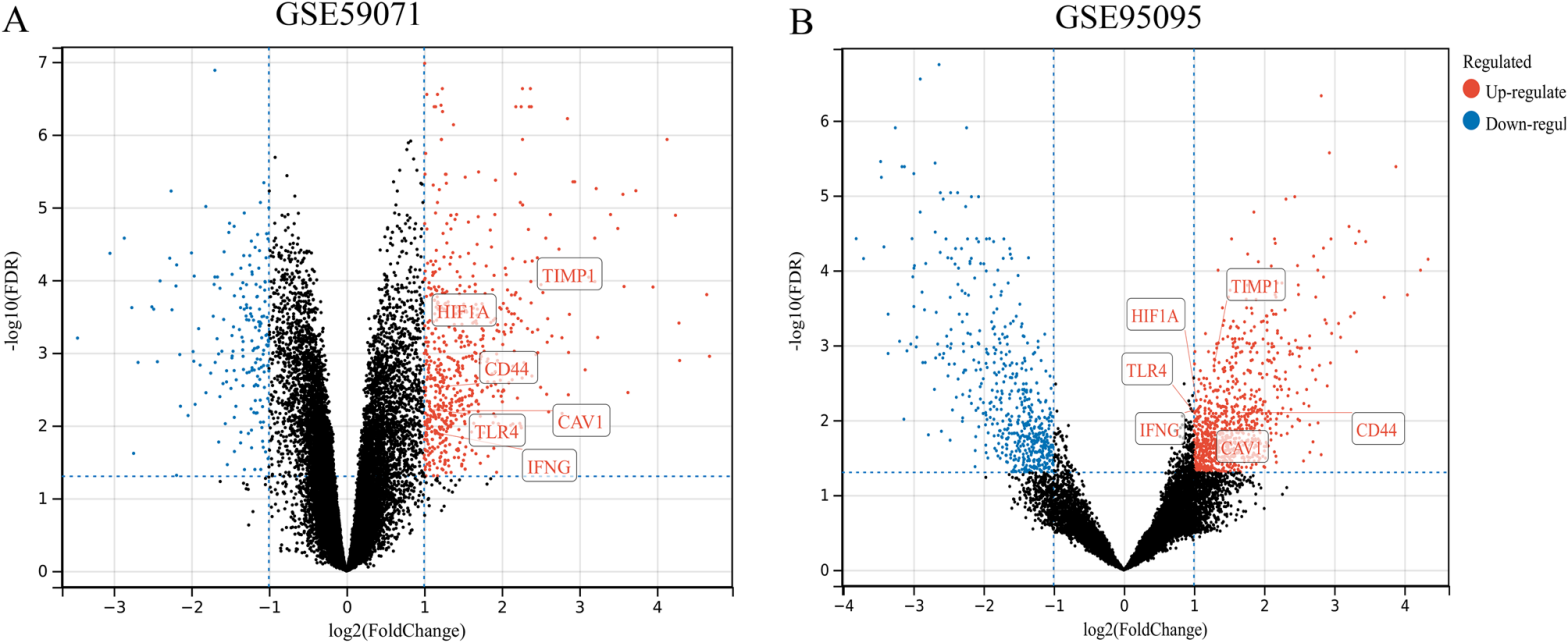
Validation of expression of key CD-FRGs in CD. (**A,B**) DEGs from the GSE59071 and GSE95095 datasets are shown, respectively. red dots represent up-regulated genes, blue dots represent down-regulated genes, and black dots indicate genes without any significant differences shown.

We selected two additional microarray datasets (GSE59071 and GSE95095) for validation to determine whether key CD-FRGs are differentially expressed in the CD datasets. A total of 751 DEGs were found in GSE59071 and 11,480 DEGs were found in GSE95095 (Fig. 3A,B). In the datasets GSE59071 and GSE95095, the 6 previously screened key CD-FRGs were validated to be upregulated DEGs.

### Functional enrichment of DE-FRGs and key CD-FRGs

**Figure 4:**
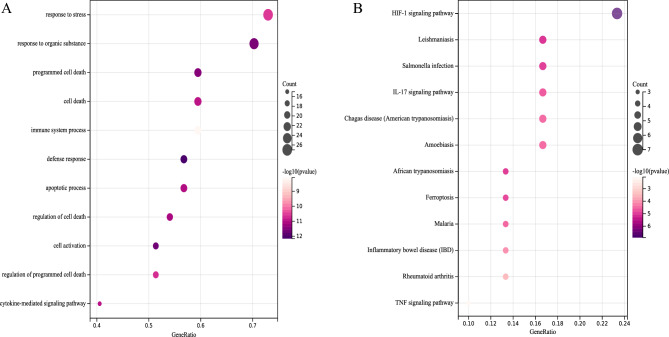
Functional enrichment analysis for DE-FRGs associated with CD. (**A**) Bubble diagram showing DE-FRG enriched with GO terms. (**B**) Bubble diagram showing DE-FRG enriched with KEGG pathways^22–24^. The darker the color and the larger the bubbles, the more significant the difference.

**Figure 5:**
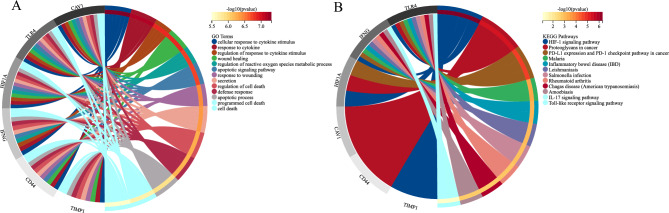
Functional enrichment analysis of key CD-FRGs related to CD. (**A**) GO terms of key CD-FRGs. (**B**) KEGG enrichment^22–24^ of key CD-FRGs. Genes are linked by colored bands to their assigned pathway terms and sorted according to the observed log10 *P* values, which are shown in decreasing intensity of the red squares next to the selected genes.

For predicting the biological functions of DE-FRGs, functional enrichment analysis was performed. GO analysis indicated that DE-FRGs were enriched mainly in programmed cell death, immune system process, apoptotic process and regulation of cell death (Fig. 4A); whereas KEGG pathway analysis indicated DE-FRGs were significantly enriched in HIF-1 signaling pathway, IL-17 signaling pathway, Ferroptosis and IBD (Fig. 4B). GO analysis revealed that key CD-FRGs were enriched significantly in regulation of reactive oxygen species metabolic process, programmed cell death and apoptotic process (Fig. 5A); whereas KEGG pathway analysis indicated key CD-FRGs were significantly enriched in HIF-1 signaling pathway and IBD (Fig. 5B). The results of functional enrichment analysis obtained for GO terms or KEGG revealed that these CD genes were associated with either programmed cell death or IBD.

### Distribution of immune-infiltrated cells

**Figure 6:**
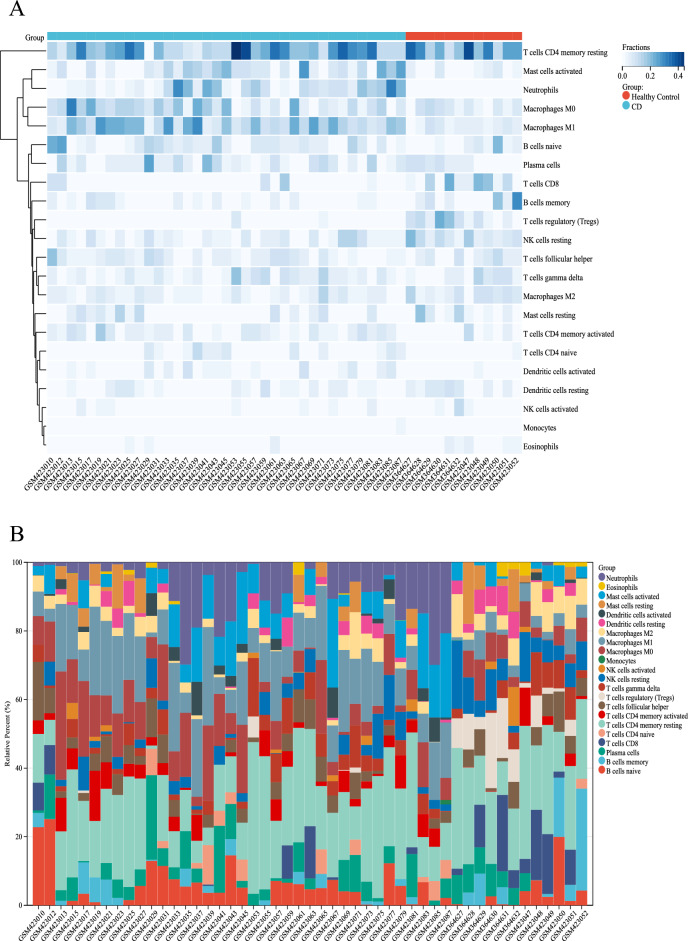
Infiltration of immune-related cells in CD and healthy control samples. (**A**) Amount of immune cells in each sample. (**B**) Relative percentages of 22 immune cell subpopulations from CD samples in the GSE16879 dataset.

Screening of the microarray with CIBERSORT inverse convolution method at *P* < 0.05 resulted in 12 healthy intestinal tissues and 37 CD intestinal tissue groups on the heat map. Memory CD4 + T cells activated, M1 macrophages, M2 macrophages, Mast cells activated and Neutrophils were all more abundant in intestinal tissues of patients with CD than in healthy controls, while CD8 + T cells, regulatory T cells (Tregs), Eosinophils and resting NK cells were less expressed in the intestinal tissues of CD patients than in the healthy control group (Fig. 6A). The distribution of the 22 immune cells in each sample was detailed in Fig. 6B.

### Analysis of the correlation and differences in immune-infiltrated cells

**Figure 7:**
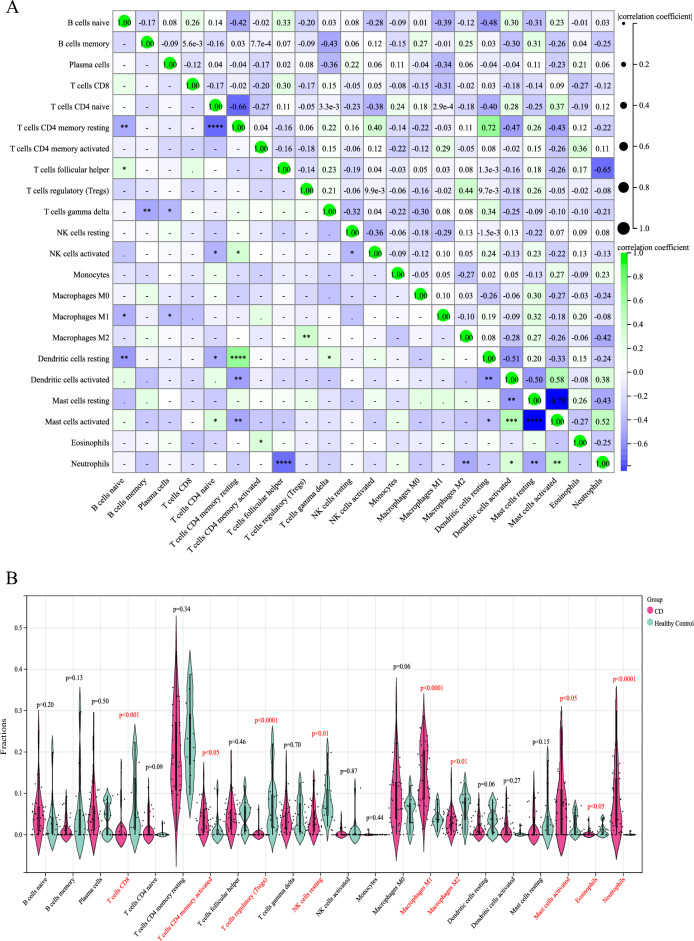
Differences and correlation analysis of immune cells in CD. (**A**) Correlation analysis of immune cells in CD. Green indicates positive correlation, blue indicates negative correlation; the higher the absolute value, the stronger the correlation between immune cells. (**B**) Violin diagram displaying the proportion of differences between healthy and CD samples for each immune cell type; light green corresponds to healthy samples and pink to CD samples, *P* < 0.05.

A positive correlation was found between memory resting CD4 + T cells and resting Dendritic cells (r = 0.72, *p* < 0.0001), activated Mast cells and activated dendritic cells (r = 0.58, *p* < 0.001), as well as activated Mast cells and Neutrophils (r = 0.52, *p* < 0.01) (Fig. 7A). Instead, a negative correlation was detected between naïve CD4 + T cells and memory resting CD4 + T cells (r =  − 0.66, *p* < 0.0001), follicular helper T cells and Neutrophils (r =  − 0.65, *p* < 0.0001), activated dendritic cells and resting dendritic cells (r =  − 0.51, *p* < 0.01), as well as Mast cells resting and Mast cells activated (r =  − 0.78, *p* < 0.0001) (Fig. 7A).

The differences in immune-infiltrated cells were shown by violin plots between the intestinal tissues of healthy individuals and CD patients and were statistically significant at *p* < 0.05. Activated memory CD4 + T cells, M1 macrophages, M2 macrophages, Mast cells activated and Neutrophils were differentially elevated in the intestinal tissues of CD patients, in contrast to CD8 + T cells, Tregs, Eosinophils and resting NK cells (Fig. 7B).

### Correlation between key CD-FRGs and immune-infiltrated cells in CD

**Figure 8:**
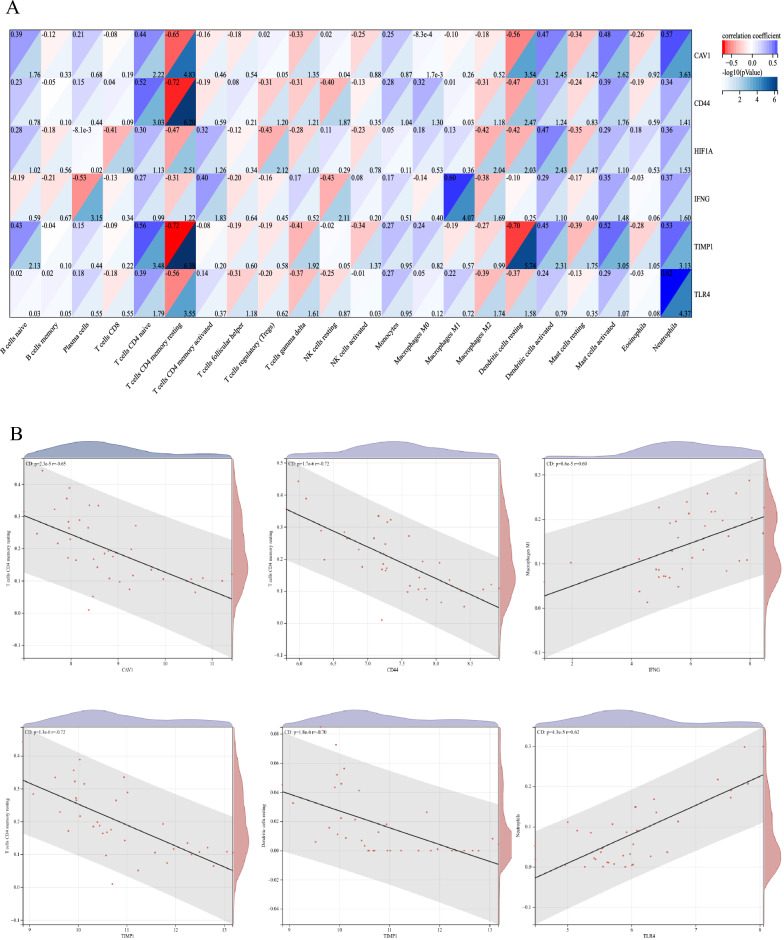
Correlation between key CD-FRG and immune-infiltrated cells in CD. (**A**) The darker the blue hue, the smaller the *P* value. (**B**) Correlation analysis of key CD-FRG and immune-infiltrated cells, *P* < 0.05.

The correlation between key CD-FRGs and immune-infiltrated cells in CD, which differed between CD and healthy controls, was performed by Spearman correlation (Fig. 8). Memory resting CD4 + T cells exhibited a negative correlation with *CAV1* (r =  − 0.65, *p* = 2.3e − 5). Memory resting CD4 + T cells displayed a negative correlation with *CD44* (r =  − 0.72, *p* = 1.7e − 6). Memory resting CD4 + T cells showed a negative correlation with *TIMP1* (r =  − 0.72, *p* = 1.3e − 6). Neutrophils exhibited a positive correlation with *TLR4* (r = 0.62, *p* = 4.3e − 5). M1 macrophages exhibited a positive correlation with *IFNG* (r = 0.60, *p* = 8.6e − 5). Resting dendritic cells displayed a negative correlation with *TIMP1* (r =  − 0.70, *p* = 1.8e − 6). Accordingly, these genes were closely correlated to the immune-infiltrated cells in CD.

## Discussion

Bioinformatics has not only helped to broaden the field of complicated multigene diseases, but has also helped to determine several genes that cause CD, which has provided novel insights into CD pathogenesis. In the present study, total of 37 DE-FRGs were found to be significantly expressed in CD intestinal tissues, whereas 6 key CD-FRGs were found, but further studies are still needed to investigate how or to what extent they participate in the pathogenesis of CD. Functional enrichment analysis of GO terms indicated that DE-FRGs were enriched mainly in programmed cell death, immune system process, apoptotic process, and regulation of cell death. KEGG pathway analysis indicated that DE-FRGs were mainly enriched in HIF-1 signaling pathway, IL-17 signaling pathway, Ferroptosis and IBD. Among the PPI network of DE-FRGs, high scores were obtained for 6 of the 37 genes (*CAV1, CD44, HIF1A, IFNG, TIMP1* and *TLR4*) in the 6 algorithms of cytoHubba. GO terms analysis revealed that these 6 key CD-FRGs were highly enriched in regulation of reactive oxygen species metabolic process, programmed cell death and apoptotic process, as well as in KEGG of HIF-1 signaling pathway and IBD. Defective apoptosis has long been thought to be associated with IBD, and the cause of CD pathogenesis is thought to be an excessive T-cell response due to an intrinsic defect in the T-cell apoptosis pathway^26^. Impaired regulation of cell death will disrupt the intestinal epithelial cell barrier and trigger a range of intestinal diseases, including CD^27^. The important role of IL-17 signaling pathway in CD has been demonstrated by numerous studies^6,28^. It has been demonstrated that HIF-1α stabilizers can be used to treat IBD^29^. These results for both GO terms and the KEGG pathway indicated that the DE-FRGs or key CD-FRGs found in the present study may be involved inin the progression of CD through the above pathways.

Caveolin-1 (*CAV1*) is a scaffold structural protein of caveolae involved in the regulation of angiogenesis, cellular signaling pathways and inflammation mediated by endothelial cells^30^. Caveolin-1 has been shown to inhibit fibroblast autophagy by regulating Sequestosome 1, which decreases intestinal fibrosis in CD^31^. As a suppressor of ferroptosis, *CAV1* has been shown to indicate ferroptosis in acute immune-mediated liver injury by attenuating nitrogen stress^32^. Therefore, it is speculated that CAV1 may affect intestinal fibrosis in CD by alleviating ferroptosis, but further studies are still needed to validate it.

*CD44* (CD44 Molecule) is a cell surface glycoprotein which has long been researched as a cancer molecule owing to its vital function in the biological activities of normal cells and in the pathological actions of cancer cells (e.g., cell proliferation, adhesion, and migration)^33^. Liu et al. revealed that *CD44* expression inhibited ferroptosis in cancer cells in an OTUB1-dependent manner^34^. It has been suggested that the absence of specific *CD44* variable splice exons in macrophages prevents CD^35^. It remains to be investigated whether *CD44*, a ferroptosis inhibitor, could exacerbate CD by reducing ferroptosis in CD.

Hypoxia-inducible factor 1-alpha (*HIF1A*) serves as a transcription factor that modulates cellular adaptation to hypoxia levels and provides support for development and function of the intestinal barrier^36^. The study by Mimouna et al. demonstrated that co-activation of autophagy and *HIF1A* expression may be a new approach to address adherent invasive E. coli (AIEC) infection in CD patients^37^. *HIF1A* has been shown to be not only a driver^38^ but also an inhibitor^39^ of ferroptosis. Thus, it is crucial to maintain the balance of *HIF1A* in CD to control ferroptosis in CD..

Interferon gamma (IFN-γ, namely *IFNG*) is a pro-inflammatory factor which regulates numerous immune-related genes^40^. Interferon γ (IFN-γ) released from CD8 + T cells impairs the uptake of cystine by tumor cells, thereby promoting lipid peroxidation and ferroptosis in tumor cells^41^. *IFNN* has long been thought to be a driver of inflammatory factors in CD and to exacerbate its course^42,43^, and coupled with its driving role in ferroptosis, we speculated that it may promote CD by exacerbating ferroptosis.

Tissue inhibitor of metalloproteinase 1 (*TIMP1*) is one of the four members of the glycoproteome (TIMP1-4), whose main feature is to mediate extracellular matrix turnover. Since the main cellular sources of TIMP-1 are macrophages and fibroblasts, regulation of these cells is essential for the prevention of colonic fibrosis in patients with CD^44,45^. Shi et al. demonstrated that silencing *TIMP1* inhibited ferroptosis in cardiac microvascular endothelial cells through downregulation of transferrin receptor 1 (TFR-1)^46^. However, whether *TIMP1* can be involved in CD progression by affecting ferroptosis in fibroblasts remains undescribed and needs to be further explored.

Toll-like receptor 4 (*TLR4*) is thought to play a key role in the pathogenesis of IBD and has been identified as an effective target for the treatment of IBD^47,48^. It has been demonstrated that knockdown of *TLR4* significantly improves left ventricular remodeling in heart failure by inhibiting ferroptosis in cardiomyocytes^49^. Therefore, we venture to speculated that *TLR4* may be able to exacerbate CD by promoting ferroptosis in intestinal cells.

Abnormal immune cell infiltration is a hallmark of CD^50^. Abnormal infiltration of immune cells is closely related to ferroptosis in intestinal tissues, and a study by Tang et al. found that intervention with OTSSP167, a selective inhibitor of MELK, significantly inhibited ferroptosis in intestinal tissues and suppressed macrophage infiltration and M1 polarization, thereby reducing the secretion of pro-inflammatory factors^51^. Earlier studies have shown that soluble caveolin-1-Ig inhibits T cell proliferation and cytokine production in response to recall antigen or alloantigen-presenting cells (APC), and in combination with our study, *CAV1* was negatively correlated with memory resting CD4 + T cells, suggesting that *CAV1* may be involved in CD progression by inhibiting T cell proliferation^52^.*CD44* has been shown to block apoptosis of CD4 + T cells that activate colitis^35,53^. The inhibitor of ferroptosis, *CD44*, was negatively correlated with memory resting CD4 + T cells in the present study, which may suggest that *CD44* exacerbates CD in the intestine by inhibiting apoptosis or ferroptosis of CD4 + T cells. It has been demonstrated that the microenvironment of IFN-γ polarizes macrophages and induces intestinal Epithelial-mesenchymal transition (EMT) affecting CD fibrosis^54^, which in addition combined with the positive correlation of *IFNG* with macrophage M1 in our study may suggest that *IFNG* could be involved in the pathogenesis of CD through macrophages. One study showed that the anti-inflammatory factor interleukin-10 can act on dendritic cells and induce their production of *TIMP1*^55^, with our study, this suggests that *TIMP1* and dendritic cells may interact to affect CD. Li et al. found that ferroptosis coordinates neutrophil recruitment to damaged myocardium by promoting neutrophils adhesion to coronary endothelial cells via the TLR4/TRIF/I type IFN signaling pathway^56^, and combined with the positive association of TLR4 and neutrophils in our study, suggests that *TLR4* may affect CD by regulating ferroptosis of neutrophils in the intestine. However, how *HIF1A* mediates ferroptosis causing CD through these immune infiltrated cells still needs to be explored. Experiments with these CD-FRGs specific immune cell knock-out may contribute to unraveling the underlying mechanisms in the future.

There are also some limitations in this study. First, it was a secondary mining and analysis of previously published datasets. Thus, the results of the experiments may vary from the findings of earlier experiments, probably due to bias in the data analysis because of a small sample size or other reasons. Second, since the CIBERSORT deconvolution algorithm relies on limited genetic data, different diseases have variability in susceptibility factors and disease phenotypes, which will lead to inaccurate results. Despite this, our study could still provide compelling evidence and new research ideas for further investigation of the role of these immune-infiltrated cells or immune-related genes in ferroptosis of CD.

## Conclusion

Taken together, immune cells with abnormal infiltration can be implicated in CD due to ferroptosis. This study identified 6 key CD-FRGs that may be key biomarkers of ferroptosis in CD; they include *CAV1, CD44, HIF1A, IFNG, TIMP1* and *TLR4*. These findings suggest that the immune response is critical in CD caused by ferroptosis through the interaction between key CD-FRGs and immune infiltrating cells.

## Data Availability

The datasets generated and/or analyzed during the current study are available in the [GEO] repository, [https://www.ncbi.nlm.nih.gov/geo/query/acc.cgi?acc=GSE16879/GSE59071/GSE95095].
